# Immunometabolic Regulation of Neuroinflammation in Retinitis Pigmentosa: Roles of Microglia, Müller Glia, and Regulated Cell Death

**DOI:** 10.3390/biom16030364

**Published:** 2026-02-28

**Authors:** Yijing Yang, Pai Zhou, Ying Deng, Qinghua Peng

**Affiliations:** 1 Faculty of Traditional Chinese Medicine, Hunan University of Chinese Medicine, Changsha 410208, China; 004761@hnucm.edu.cn (Y.Y.); zhoupai2020@gmail.com (P.Z.); 2 Institute of Ophthalmology and Otolaryngology of Chinese Medicine, Changsha 410208, China; 3 Changsha Centre for Innovation in Traditional Chinese Medicine Techniques for the Prevention and Treatment of Retinal Diseases and Visual Function Protection, Changsha 410208, China

**Keywords:** retinal degeneration, neuroinflammation, microglia, p53

## Abstract

Chronic neuroinflammation is increasingly implicated in the progression of neurodegenerative diseases, yet the mechanisms linking metabolic stress, innate immune activation, and neuronal vulnerability remain incompletely defined. Retinitis pigmentosa (RP), despite its genetic heterogeneity, exhibits convergent inflammatory and metabolic alterations during disease progression, providing a useful model for studying immune-mediated neurodegeneration. This review summarizes current evidence from experimental models of retinal degeneration and human retinal studies to examine how sustained neuroinflammation is established in RP. We focus on the coordinated roles of retinal microglia and Müller glia in sensing photoreceptor stress and shaping the inflammatory microenvironment. Microglia are activated early in disease and contribute to progression through inflammatory signaling, phagoptosis, metabolic adaptation, and inflammasome-associated pathways. Müller glia, in turn, modulate metabolic homeostasis and propagate inflammatory signals across retinal layers. We also discuss how stress-responsive regulatory pathways, including p53-associated signaling, influence redox balance, iron handling, and inflammatory persistence without acting as primary apoptotic drivers. Together, these findings support a model in which chronic immunometabolic dysregulation contributes to retinal degeneration and highlight inflammation-related processes as potential targets for mutation-independent therapeutic strategies.

## 1. Introduction

Neuroinflammation is increasingly recognized as a fundamental driver of chronic neurodegenerative disease, challenging the long-standing view that neuronal loss is governed primarily by cell-autonomous death programs. Over the past decade, work across the central nervous system has shown that sustained activation of innate immune pathways is not merely a late consequence of degeneration but actively shapes disease trajectory from early stages onward [1,2]. Sustained microglial engagement, metabolic stress signaling, and self-reinforcing inflammatory loops have emerged as defining features of progressive neurodegeneration [1,3], prompting a shift toward disease models in which immunometabolic coupling shapes vulnerability to determine neuronal vulnerability.

Within this evolving framework, RP provides a particularly informative model of chronic neurodegeneration. Although RP is initiated by a wide spectrum of genetic mutations that disrupt photoreceptor-specific processes [4,5], disease progression converges on a shared set of pathological features. Across genotypes, retinal degeneration is accompanied by sustained inflammatory activation and metabolic stress within the retinal microenvironment [6,7]. The retina, as an immune-privileged neural tissue enriched in resident innate immune cells—most notably microglia—therefore offers a system in which to examine how chronic cellular stress is translated into persistent neuroinflammatory signaling [8,9].

Historically, photoreceptor degeneration in RP was interpreted largely through the lens of apoptosis. Early observations of DNA fragmentation and caspase activation supported the notion that classical apoptotic pathways constituted a final common route of cell death [5]. However, this view has proven incomplete. Subsequent studies documented widespread deviations from canonical apoptosis, including caspase-independent degeneration, atypical nuclear morphology, and profound disturbances in metabolic and redox homeostasis [10,11,12]. Consistent with these observations, pharmacological inhibition of apoptotic executioners frequently fails to preserve photoreceptor survival, indicating that ferroptosis/inflammasome signaling exert substantial influence over disease progression [11,12].

These findings align with broader immunological evidence demonstrating that regulated cell death pathways are tightly coupled to inflammatory signaling. Non-apoptotic forms of regulated cell death, including ferroptosis and inflammasome-associated pyroptotic signaling, generate immunogenic stress cues that actively engage innate immune cells [13,14,15]. In this light, cell death is not solely a terminal event but also a source of inflammatory amplification, capable of sustaining immune activation and exacerbating tissue injury.

Against this background, the tumor suppressor p53 occupies a paradoxical position in RP research. Genetic ablation studies established that photoreceptor apoptosis can proceed independently of p53, particularly in rapidly degenerating experimental models [16,17]. These results fostered the prevailing assumption that p53 plays a minimal role in retinal degeneration. However, such conclusions derive from a narrow focus on apoptotic execution and do not account for the broader regulatory functions of p53 in oxidative stress control, metabolic adaptation, iron homeostasis, and innate immune signaling [1,18,19,20]. However, much of the RP evidence comes from global p53 ablation, limiting cell type inference.

Indeed, accumulating evidence from immune and glial biology positions p53 as a stress-responsive modulator at the interface between metabolism and inflammation. Rather than acting as a binary switch for cell death, p53 influences inflammatory thresholds, redox balance, and the persistence of immune activation across multiple cell types. From this perspective, p53 independence of apoptotic execution does not preclude a substantial role in shaping the inflammatory and metabolic context in which retinal degeneration unfolds. While previous reviews have examined microglial activation, complement signaling, or individual regulated cell death pathways in retinal degeneration, the present synthesis advances the field by proposing an integrated immunometabolic framework centered on stress response coordination across retinal cell types. Rather than reviewing each pathway independently, we examine how ferroptotic stress, inflammasome priming, and glial metabolic remodeling may converge through shared regulatory nodes.

In this review, we synthesize evidence from experimental models of retinal degeneration, human retinal transcriptomic studies, and broader neuroimmunological research to re-evaluate the role of p53 in RP. We propose that p53 functions as a context-dependent regulator of inflammatory and metabolic stress rather than a direct mediator of neuronal apoptosis [21,22,23]. We focus on retinal microglia as a central innate immune hub that translates photoreceptor-derived stress signals into sustained neuroinflammation [24,25], and we integrate ferroptosis- and pyroptosis-related signaling into a unified disease-relevant framework that amplifies degenerative progression [13,26,27]. By positioning RP as a model of immune-driven neurodegeneration, this synthesis aims to connect retinal biology with broader disease mechanisms relevant across chronic neurodegenerative disorders.

### Literature Search Strategy and Scope

This review is based on a structured but non-systematic search of the literature conducted in PubMed/MEDLINE, Web of Science, and Scopus. The primary focus was on studies published between 2000 and 2025, with inclusion of earlier foundational work where mechanistically essential for contextual interpretation. Representative search terms included combinations such as “retinitis pigmentosa” AND “microglia,” “retinitis pigmentosa” AND “Müller glia,” “retinal degeneration” AND “ferroptosis,” “retina” AND “NLRP3 inflammasome,” “p53” AND “retina” OR “microglia,” and “phagoptosis” AND “retina.”

Inclusion criteria prioritized experimental retinitis pigmentosa animal models, studies involving human retinal tissue or transcriptomic datasets, and mechanistic investigations directly addressing immunometabolic pathways. High-quality cross-central nervous system (CNS) studies were included when extrapolation to the retina was explicitly justified. Exclusion criteria comprised purely descriptive reports lacking mechanistic insight, non-peer-reviewed sources, and studies unrelated to retinal or CNS immune–metabolic coupling. The final literature search was conducted in January 2026. Only studies published in English were included. Primary experimental studies (animal models, human tissue analyses, and mechanistic investigations) were prioritized for evidentiary synthesis. Previous reviews were consulted selectively for contextual framing and identification of seminal references but were not used as primary sources of mechanistic evidence.

This work represents a narrative, hypothesis-generating synthesis rather than a systematic review; accordingly, it does not aim to exhaustively catalog all published studies but instead integrates representative and mechanistically informative evidence to support conceptual development.

## 2. Microglia–Müller Glia Axis as the Cellular Substrate of Retinal Neuroinflammation

For many years, inflammatory changes observed in retinitis pigmentosa were interpreted primarily as secondary consequences of tissue injury. Activated microglia and reactive Müller glia were described as parallel histopathological features accompanying photoreceptor degeneration, rather than as coordinated drivers of disease progression. Early pathological studies emphasized the accumulation of activated microglia in degenerating retinas and the hypertrophic response of Müller glia as characteristic markers of retinal damage [6,8,9,28,29], reinforcing the view that glial activation merely reflected ongoing neuronal loss. However, this interpretation has become increasingly difficult to reconcile with accumulating experimental and human evidence. The temporal emergence, spatial organization, and sustained activity of retinal glia indicate that inflammatory responses are not simply epiphenomena but integral components of the degenerative process. Rather than acting in isolation, microglia and Müller glia engage in coordinated and persistent interactions that reshape the retinal microenvironment, creating a context in which neuronal stress is amplified and propagated.

Although microglia and Müller glia are often grouped together under the umbrella term “retinal glia”, this classification obscures fundamental biological distinctions that are central to understanding retinal neuroinflammation. Microglia derive from yolk sac progenitors and colonize the retina early in development, where they function as specialized innate immune sentinels capable of rapidly sensing and responding to cellular stress [8,24]. Müller glia, by contrast, arise from the neuroectoderm and form a unique radial scaffold spanning the entire thickness of the retina, providing structural support, metabolic coupling, and regulation of the neuronal microenvironment [23,30,31,32].

These developmental and anatomical differences are not merely descriptive but carry important functional implications during retinal degeneration. Microglia are positioned to initiate early immune responses to photoreceptor stress through surveillance, migration, and inflammatory activation. Müller glia, in contrast, are ideally suited to integrate these immune signals over space and time, redistributing metabolic and inflammatory cues across retinal layers and sustaining their impact. This division of labor establishes glia axis that links localized immune activation to widespread retinal inflammation. The glia axis thus provides a cellular substrate through which transient stress signals are converted into persistent inflammatory remodeling of the retina, setting the stage for progressive neuronal dysfunction and loss.

### 2.1. Microglia as Early Innate Immune Sensors in RP

Evidence from multiple genetic models of retinitis pigmentosa—including rd1, rd10, P23H rhodopsin, and CNGB1-deficient mice—has progressively shifted attention toward microglia as early participants in disease pathogenesis. Rather than emerging only after widespread neuronal loss, microglial activation is consistently detected at initial disease stages, often before overt photoreceptor apoptosis and measurable retinal thinning become apparent [8,9,28]. This temporal relationship argues against a purely reactive role and instead suggests that immune activation is intertwined with the earliest phases of retinal degeneration.

Concomitant with their early activation, microglia undergo pronounced morphological and functional remodeling. Cells transition from a highly ramified surveillant phenotype to a more amoeboid and motile state, reflecting a shift toward active immune engagement. In parallel, microglia redistribute from inner retinal layers toward the outer nuclear layer, where metabolically stressed photoreceptors are concentrated, and upregulate inflammatory mediators, phagocytic markers, and complement receptors [9,28,33]. This coordinated program of migration and phenotypic transformation positions microglia as the earliest innate immune sensors of photoreceptor distress in RP.

Temporal analyses further refine the role of microglia in early RP pathogenesis. Microglial recruitment is observed at stages when a substantial proportion of photoreceptors remain structurally preserved, arguing against a model in which microglia are mobilized merely to clear cellular debris. Instead, these cells appear capable of sensing early, sublethal neuronal dysfunction and responding by initiating inflammatory programs that subsequently influence the course of degeneration [8,28]. Such timing places microglia not at the end of the degenerative cascade, but at its beginning, positioning them as active contributors to disease initiation rather than passive responders to late-stage tissue breakdown.

### 2.2. Immune-Mediated Neuronal Loss and Microglial Phagoptosis

A direct pathological consequence of microglial activation in RP is immune-mediated neuronal loss through phagoptosis, defined as the engulfment of stressed yet viable neurons [28,34]. In degenerating retinas, activated microglia infiltrate the outer nuclear layer and establish intimate contact with photoreceptors that have not initiated intrinsic death programs [33,35,36]. This behavior distinguishes phagoptosis from debris clearance and implicates microglia as active participants in neuronal elimination.

Complement signaling plays a central role in this process. Upregulation of complement components and complement receptors enhances opsonization of stressed photoreceptors and facilitates their recognition by microglia [33,35,36]. Consistent with a causal role, genetic or pharmacological disruption of complement pathways, microglial recruitment, or phagocytic machinery delays photoreceptor loss and preserves retinal structure and function across multiple RP models [7,33,35,37]. These findings indicate that neuronal elimination in RP is not dictated solely by intrinsic apoptotic programs. Instead, immune-mediated engulfment constitutes an active driver of degeneration, highlighting a mode of neuronal loss in which inflammation and cell death are mechanistically inseparable.

### 2.3. Müller Glia as Metabolic and Inflammatory Integrators

Whereas microglia dominate the initiation of immune responses, Müller glia occupy a distinct but equally critical position in shaping the metabolic and inflammatory landscape of the degenerating retina. Under physiological conditions, Müller glia serve as central homeostatic regulators, maintaining extracellular potassium and glutamate balance, supplying metabolic substrates to neurons, and contributing to antioxidant defense mechanisms essential for retinal stability [23,30,31]. These functions position Müller glia as key buffers against metabolic stress in the healthy retina.

During the early stages of RP, Müller glia initially mounts compensatory responses aimed at preserving tissue homeostasis. Activation of antioxidant pathways and reinforcement of metabolic coupling with photoreceptors represent adaptive efforts to stabilize neuronal function under conditions of emerging stress [20]. At this stage, Müller glial reactivity may serve a protective role, attempting to counterbalance the metabolic disturbances triggered by photoreceptor dysfunction.

With sustained exposure to inflammatory cytokines, oxidative stress, and metabolic imbalance; however, this adaptive state progressively gives way to reactive gliosis. This transition is characterized by cellular hypertrophy, increased expression of glial fibrillary acidic protein, cytoskeletal remodeling, and widespread transcriptional reprogramming [3,23,31]. In contrast to the rapid and highly dynamic activation of microglia, Müller glial gliosis develops more slowly but persists throughout disease progression, reshaping the retinal environment over extended time scales.

Reactive Müller glia increasingly contribute cytokines, chemokines, and extracellular matrix components that alter local tissue architecture and intensify neuronal stress [21]. The crucial change, therefore, is not merely anatomical but metabolic and inflammatory. As buffering capacity erodes, Müller glia move from absorbing stress to redistributing it, transforming chronic metabolic imbalance into a persistent pro-inflammatory environment that supports ongoing degeneration.

### 2.4. Bidirectional Microglia–Müller Glia Crosstalk as an Amplification Loop

Microglia and Müller glia do not operate as parallel responders. Their interaction is mechanistically coupled through reciprocal signaling events that convert localized immune activation into sustained retinal inflammation. Activated microglia release pro-inflammatory cytokines and reactive oxygen species that directly induce Müller glial gliosis and metabolic reprogramming. Exposure to these mediators enhances Glial fibrillary acidic protein (GFAP) expression and shifts Müller glial transcriptional programs toward chemokine production. In turn, reactive Müller glia secrete chemotactic signals such as Chemokine (C-C motif) ligand 2 (CCL2) and other inflammatory mediators that promote further microglial recruitment and activation [29,38,39].

This sequence—microglial cytokines and Reactive oxygen species (ROS) driving Müller activation, followed by Müller-derived chemokines sustaining microglial engagement—illustrates the operational logic of the circuit. Rather than re-labeling this interaction as a “self-reinforcing loop,” the key point is functional: each cell type modifies the state of the other in ways that prolong inflammatory tone.

Comparable immune–glial coupling has been documented in other neurodegenerative contexts, indicating that the retina follows broader neuroimmunological principles rather than a tissue-specific anomaly [1,2,3]. Human RP retinas demonstrate the same coordinated pattern, with activated microglia/macrophages accumulating in the outer retina alongside extensive Müller gliosis and GFAP upregulation [7,40,41]. Notably, microglial activation extends beyond zones of overt neuronal loss, consistent with a sustained inflammatory state that cannot be explained solely by debris clearance.

### 2.5. p53-Regulated Stress Integration Within the Glial Inflammatory Axis

In this self-amplifying glial network, p53-regulated stress signaling introduces an additional layer of immunometabolic coordination. In microglia, p53 influences cellular tolerance to oxidative stress, inflammatory polarization, and metabolic adaptation through interactions with Nuclear factor kappa B (NF-κB) signaling and mitochondrial stress pathways [1,19,20,42]. These processes bias microglial behavior toward sustained inflammatory activation rather than timely resolution.

In Müller glia, emerging evidence points to a parallel role for p53-associated stress programs in shaping long-term glial behavior. p53-linked pathways have been implicated in metabolic reprogramming, persistence of gliosis, and continued production of inflammatory mediators under conditions of chronic stress [22,43,44]. Although cell-type-specific manipulation of p53 in Müller glia remains limited, available data collectively suggest that p53 signaling favors a shift from supportive homeostatic functions toward maladaptive inflammatory states. Through this mechanism, p53 may reinforce the stability of the microglia–Müller glia inflammatory axis, thereby contributing to the chronicity of retinal neuroinflammation.

Single-cell transcriptomic studies further identify enrichment of innate immune pathways, stress response programs, and p53-associated gene networks within glial populations from degenerating human retinas [21,22,43,45]. The close overlap between these signatures and findings from experimental models supports the idea that microglia-driven inflammatory mechanisms are conserved across species and disease stages.

**Figure 1:**
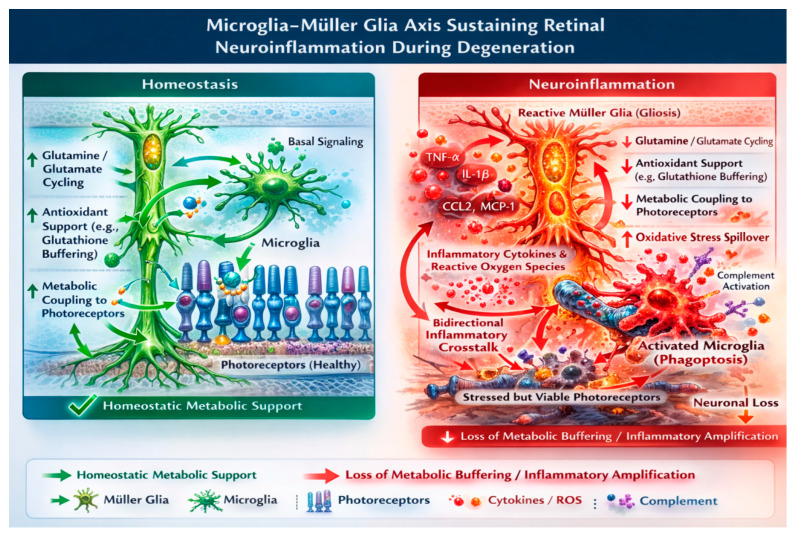
Microglia–Müller glia axis sustaining retinal neuroinflammation during degeneration. Schematic illustrating bidirectional communication between microglia and Müller glia during retinal degeneration. Genetic mutations and oxidative–metabolic stress (Glutamine/glutamate cycling, Antioxidant support, and Metabolic coupling to photoreceptors) in photoreceptors trigger the oxidative stress spillover, including extracellular ATP, oxidized lipids, and iron dysregulation. These signals recruit and activate microglia, promoting inflammatory cytokines and reactive oxygen species. Activated microglia, in turn, induce Müller glial activation and reactive gliosis. BioRender: https://www.biorender.com (Figures created using the BioRender web-based platform; accessed January 2026.); Adobe Illustrator: Adobe Illustrator 2021 (Version 25.4.1, Adobe Inc., San Jose, CA, USA).

These findings support a model in which retinal neuroinflammation in RP is driven by the glia axis that acts as an innate immune–glial amplification unit. By combining chronic neuronal stress with immune activation and metabolic dysfunction, this axis provides a cellular basis for ongoing inflammation and progressive neurodegeneration (Figure 1).

Gliosis is accompanied by a decline in glutamine–glutamate cycling, impaired antioxidant substrate delivery (including reduced glutathione buffering capacity), and weakening of metabolic coupling to photoreceptors. The resulting loss of metabolic buffering increases oxidative stress spillover and lowers the threshold for ferroptotic injury in vulnerable neurons. Through this reciprocal amplification, the microglia–Müller glia axis converts localized stress into sustained, layer-spanning neuroinflammation and heightened ferroptotic susceptibility during retinal degeneration.

## 3. Microglia-Specific Mechanisms Driving Neuroinflammation in RP

A mechanistic understanding of retinal neuroinflammation requires close examination of microglial behavior at the earliest stages of disease. In retinitis pigmentosa, microglial activation is consistently observed as an early event, emerging before extensive photoreceptor loss or overt structural remodeling of the retina [24,25,45]. Across genetically diverse models, microglial reactivity precedes large-scale neuronal degeneration, challenging the view that immune activation is merely secondary to cell death. Instead, the temporal sequence of these responses positions microglia at the initiation of inflammatory signaling cascades, suggesting that they contribute to shaping the early trajectory of disease rather than functioning solely as late-stage scavengers of cellular debris.

### 3.1. Early Stress Sensing and Microglial Recruitment

Metabolic and proteostatic stress in photoreceptors is accompanied by the release of a spectrum of danger-associated molecular patterns, including extracellular ATP, oxidized lipids, mitochondrial DNA, and misfolded proteins. Rather than responding to cell death per se, microglia are equipped to sense these early distress signals through purinergic receptors, pattern-recognition receptors, and scavenger receptors, enabling them to detect neuronal dysfunction before overt degeneration occurs [24,46]. Activation of these sensing pathways initiates directed microglial migration toward the outer nuclear layer, positioning microglia in close proximity to stressed photoreceptors.

Experimental studies further illustrate the specificity of this recruitment process. In several RP models, purinergic signaling via adenosine A2A receptors promotes microglial chemotaxis and dynamic process extension, facilitating early physical interactions with photoreceptors at stages when many neurons remain viable [46]. Such findings argue that microglial recruitment reflects active surveillance of neuronal distress rather than a delayed response to cellular debris.

Single-cell transcriptomic analyses reinforce this interpretation. Recruited microglia in degenerating mouse and human retinas exhibit coordinated upregulation of stress response and innate immune gene programs, including p53-associated transcriptional networks [21,22,43,45]. These transcriptional signatures are more consistent with engagement of intrinsic stress-adaptive pathways than with passive phagocytic activation, supporting the view that microglial recruitment represents an early, regulated immune response to neuronal dysfunction.

### 3.2. Inflammatory Polarization and p53–NF-κB Crosstalk

Following their recruitment to the outer retina, microglia do not remain in a neutral surveillance state but progressively adopt a pro-inflammatory transcriptional profile. This shift is characterized by increased expression of cytokines such as TNF-α, IL-1β, and IL-6, together with chemokines that amplify immune signaling and exacerbate neuronal stress [8,25]. Such polarization marks a transition from transient immune activation toward a more persistent inflammatory phenotype.

A central regulatory feature of this transition lies in the interaction between p53 and NF-κB signaling. Although these pathways are often portrayed as antagonistic during acute cellular stress, this relationship changes under chronic conditions. Sustained oxidative and metabolic stress—defining features of retinal degeneration—favors their coordinated activation, promoting continued inflammatory signaling rather than apoptotic resolution [19,47]. In macrophages and microglia, p53 activity has been shown to stabilize NF-κB-dependent transcriptional programs, thereby maintaining inflammatory gene expression during prolonged stress exposure [48,49].

Evidence from RP models is consistent with this regulatory coupling. Interventions that reduce oxidative burden or limit iron accumulation attenuate both p53 and NF-κB signaling and are accompanied by diminished microglial activation [50]. These observations support a functional link between p53-regulated stress responses and the persistence of inflammatory microglial states, positioning p53–NF-κB crosstalk as a key mechanism sustaining inflammatory persistence in retinal degeneration.

### 3.3. Microglial Phagoptosis and Immune-Mediated Neuronal Loss

Among the microglia-centered mechanisms implicated in photoreceptor degeneration, phagoptosis has emerged as a particularly consequential process. Rather than clearing only dead cells, microglia can actively engulf neurons that are stressed yet still viable, thereby converting immune surveillance into a direct mode of neuronal elimination [34]. In degenerating retinas, activated microglia infiltrate the outer nuclear layer and selectively target photoreceptors that have not engaged intrinsic death programs, indicating that immune-mediated clearance can precede classical cell-autonomous degeneration [35].

Complement signaling plays a pivotal role in directing this process. Upregulation of complement components and their receptors enhance the opsonization of photoreceptors and promotes their recognition by microglia [35,36]. The functional importance of this pathway is underscored by interventional studies: genetic disruption or pharmacological inhibition of complement signaling or microglial phagocytic machinery consistently delays photoreceptor loss and preserves retinal structure and function [33,36]. These findings establish phagoptosis not as an epiphenomenon of degeneration, but as a causal driver of disease progression.

p53 may influence this process indirectly by shaping the inflammatory and oxidative milieu that governs neuronal vulnerability to immune clearance. In immune cells, p53 regulates scavenger receptor expression and phagocytic capacity, implicating p53-dependent stress signaling in the control of engulfment behavior during chronic inflammation [48,51]. Within the context of RP, such regulation suggests that p53 does not simply determine neuronal survival through intrinsic death pathways, but may also modulate the immune mechanisms that actively eliminate compromised photoreceptors.

### 3.4. Metabolic Reprogramming and Iron Handling in Activated Microglia

Sustained inflammatory activation in microglia is accompanied by a fundamental shift in cellular metabolism. Rather than relying predominantly on oxidative phosphorylation, activated microglia increasingly adopt a glycolytic metabolic profile to support the energetic demands of inflammatory signaling [52,53]. In RP, this transition is further amplified by chronic exposure to oxidized lipids and iron released from degenerating photoreceptors, situating metabolic reprogramming at the intersection of immune activation and tissue stress, Table 1.

Microglia are also central regulators of iron homeostasis within the nervous system, and inflammatory stimulation promotes iron uptake and intracellular retention [54]. In models of retinal degeneration, activated microglia accumulate iron and exhibit transcriptional signatures associated with oxidative stress and ferroptosis-related pathways [21,50]. Iron overload enhances reactive oxygen species generation and lipid peroxidation, thereby reinforcing inflammatory signaling and increasing neuronal vulnerability. Through this mechanism, metabolic imbalance and immune activation become tightly coupled.

p53 occupies a strategic position within this metabolic–inflammatory interface. By regulating mitochondrial function, redox balance, and antioxidant defenses under stress, p53 shapes cellular adaptation to metabolic strain [18,20]. In microglia, p53-dependent modulation of redox state and iron metabolism may favor prolonged inflammatory activation rather than timely resolution, providing a mechanistic link between metabolic stress and immune persistence during retinal degeneration.

### 3.5. Inflammasome Priming and Sub-Lytic Pyroptotic Signaling

Increasing evidence indicates that microglia in retinitis pigmentosa adopt a state of sustained inflammasome activation rather than undergoing widespread inflammatory cell death. Activated microglia display enhanced expression of NLRP3 inflammasome components and persistent IL-1β production, consistent with a primed inflammatory phenotype [55,56,57]. Although robust microglial pyroptotic lysis has not been conclusively demonstrated in RP, chronic inflammasome signaling appears sufficient to perpetuate cytokine release and immune amplification. This pattern suggests that inflammasome activity in RP may operate below the threshold required for overt cell rupture, thereby sustaining inflammation without depleting the microglial population.

p53 provides an additional regulatory layer to this process. By controlling mitochondrial stress responses, reactive oxygen species generation, and NF-κB-dependent priming of inflammasome components, p53 intersects multiple checkpoints governing inflammasome activation [47,58]. Under conditions of prolonged metabolic and oxidative stress, p53 may reduce the activation threshold of inflammasome signaling in microglia, enabling persistent cytokine release while avoiding lytic pyroptotic death. Such sub-lytic inflammasome activity offers a mechanistic explanation for how chronic neuroinflammation can be maintained in RP without exhausting the inflammatory cell pool.

Human RP retinas display sustained microglial activation, characterized by accumulation of Iba1-positive cells in the outer retina and continued expression of inflammatory mediators even at advanced disease stages [7,40]. Microglial activation extends beyond zones of obvious neuronal loss and spans multiple retinal layers, consistent with a chronic inflammatory state rather than a transient response to dying cells.

**Figure 2:**
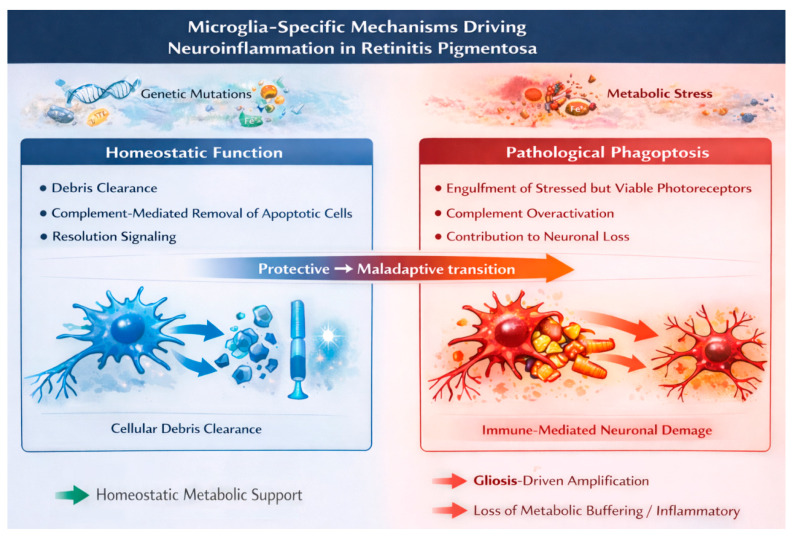
Microglia-specific mechanisms driving neuroinflammation in retinitis pigmentosa. Schematic illustrating the functional transition of retinal microglia from physiological homeostatic roles to pathological neurodegenerative activity during retinitis pigmentosa progression. The left panel depicts homeostatic microglial functions, including debris clearance, complement-mediated removal of apoptotic photoreceptors, and resolution signaling that supports tissue integrity and inflammatory control. The right panel illustrates pathological phagoptosis, characterized by complement overactivation, engulfment of stressed but viable photoreceptors, and consequent contribution to neuronal loss. Visual differentiation highlights intact neuronal silhouettes and blue arrows for protective clearance versus stressed photoreceptors and orange/red arrows for maladaptive phagoptosis. The labeled “Protective → Maladaptive transition” emphasizes the context-dependent shift in microglial function that underlies immune-mediated amplification of retinal degeneration. BioRender: https://www.biorender.com (Figures created using the BioRender web-based platform; accessed January 2026.); Adobe Illustrator: Adobe Illustrator 2021 (Version 25.4.1, Adobe Inc., San Jose, CA, USA).

These findings portray microglia as a key inflammatory hub that integrates stress signals from photoreceptors, metabolic dysregulation, and innate immune activation (Figure 2). Through processes such as inflammatory polarization, phagoptosis, metabolic reprogramming, and inflammasome priming, microglia actively promote chronic neuroinflammation and neuronal vulnerability in RP. p53-related stress response pathways intersect with all of these mechanisms, influencing microglial behavior and being strengthened by Müller glia-mediated metabolic and inflammatory feedback, providing a vital mechanistic link between cellular stress and the p53-centered inflammatory death pathway.

Under physiological conditions, microglia perform protective immune surveillance, clearing apoptotic debris and supporting tissue homeostasis. During disease progression, however, sustained stress signaling induces inflammatory polarization, metabolic reprogramming, and iron accumulation in microglia. A key pathological feature is phagoptosis, defined here as the engulfment of stressed but still viable photoreceptors that have not yet engaged intrinsic death programs. This represents a mechanistic shift from protective debris clearance to maladaptive immune-mediated neuronal elimination.

Microglial and stress response pathways in retinitis pigmentosa are inherently context dependent and should not be viewed as uniformly pathogenic. Early microglial activation supports surveillance, debris clearance, and transient neuroprotective functions before sustained inflammatory polarization emerges. Complement signaling likewise exhibits dual roles, contributing to physiological removal of apoptotic material while promoting phagoptosis under conditions of excessive activation. p53 signaling shows similar nuance, facilitating metabolic adaptation and antioxidant defense during moderate stress but potentially reinforcing inflammatory persistence when chronically engaged. These observations indicate that retinal immunometabolic responses evolve along a protective-to-maladaptive continuum, emphasizing the importance of temporal and microenvironmental context in interpreting glial activation during disease progression.

## 4. A p53-Centered Inflammatory Death Circuit Integrating Ferroptosis and Pyroptosis

Microglia-centered mechanisms help explain how neuroinflammation is initiated and sustained in retinitis pigmentosa (RP), yet an important conceptual gap remains: how are metabolic stress, regulated cell death pathways, and innate immune activation coordinated into a self-reinforcing degenerative program? Rather than presenting these processes as isolated cascades, we propose a conceptual framework in which p53 may function as a stress-responsive integrator linking ferroptosis- and inflammasome-related signaling within a broader immunometabolic network. Available evidence is consistent with a model whereby these pathways converge to support chronic retinal degeneration, although the degree of direct mechanistic coupling varies across cell types and experimental systems.

### 4.1. Ferroptosis as an Immunogenic Stress Amplifier in Retinal Degeneration

Ferroptosis is defined by iron-dependent lipid peroxidation and collapse of the glutathione–GPX4 antioxidant defense system [26,59]. Photoreceptors appear particularly vulnerable to this form of injury due to their high metabolic demand, abundance of polyunsaturated fatty acids, and sustained oxidative exposure. These intrinsic features plausibly lower the threshold for ferroptotic damage under conditions of metabolic imbalance.

Multiple RP animal models demonstrate early lipid peroxidation, iron dysregulation, and declining antioxidant capacity preceding overt photoreceptor loss [27,50]. Pharmacological inhibition of ferroptosis preserves photoreceptor viability even when classical apoptotic markers are unchanged [27,50,60]. These observations directly support ferroptotic susceptibility as a contributing mechanism in experimental RP.

Ferroptosis-associated byproducts—including oxidized lipids and iron species—are recognized activators of innate immune pathways [61]. In retinal models, such danger signals are temporally associated with microglial activation, consistent with a biochemical bridge between neuronal metabolic stress and immune engagement. While direct in vivo causal mapping remains incomplete, available data support the interpretation that ferroptosis may amplify neuroinflammation rather than merely accompany degeneration.

p53 regulates cystine transport and glutathione metabolism, notably through repression of SLC7A11 [13,60]. In degenerating retinas, p53 activation coincides with reduced antioxidant capacity and increased lipid peroxidation. These findings are consistent with a model in which p53 lowers the threshold for ferroptotic stress. However, whether p53 directly coordinates ferroptosis and immune activation in a linear causal sequence remains to be established in cell-type-specific systems.

### 4.2. Microglial Amplification of Immuno-Ferroptotic Signaling

Microglial activation is robustly documented in RP animal models and human retinal tissue, including early migration toward the outer nuclear layer and inflammatory polarization [21,50]. Iron accumulation and metabolic reprogramming signatures in activated microglia are supported by transcriptomic and histological data in experimental systems. These domains therefore rest on comparatively strong evidence.

Available data are consistent with the interpretation that lipid peroxidation products and iron released from stressed photoreceptors provide activating cues for microglia. Once activated, microglia release reactive oxygen species and pro-inflammatory cytokines, which may increase neighboring neuronal susceptibility to ferroptotic stress. This reciprocal amplification between metabolic injury and immune signaling is supported in animal models, though the precise quantitative contribution of each component remains unresolved.

p53 modulates mitochondrial function, antioxidant defenses, and iron handling in neurons and immune cells [18,20]. These roles are experimentally supported in broader neurobiology. In RP specifically, p53 activation overlaps with inflammatory and oxidative signatures, suggesting that p53 may influence how ferroptotic stress is interpreted by the immune system. However, direct evidence demonstrating p53-dependent microglial reprogramming in vivo remains limited.

### 4.3. Inflammasome Signaling and Sub-Lytic Inflammatory Persistence

Inflammasome activation—particularly NLRP3 priming and IL-1β signaling—is repeatedly observed in RP animal models and human retinal tissue [56,62]. These findings directly support the presence of inflammasome priming signatures in degenerating retinas.

Classical pyroptosis involves gasdermin-mediated membrane pore formation, cytokine release, and lytic cell death [63,64]. In RP, however, current evidence more strongly supports sustained inflammasome activation than widespread microglial lysis. Elevated inflammasome component expression and IL-1β production are documented, whereas definitive in vivo demonstration of extensive gasdermin-dependent execution remains incomplete.

Thus, available evidence is consistent with a model in which inflammasome signaling may operate in a sub-lytic mode, permitting cytokine release without overt depletion of the microglial population. The extent of gasdermin-mediated pore formation and the degree of true pyroptotic execution in RP in vivo remain areas requiring direct experimental clarification.

p53 intersects mitochondrial stress responses, reactive oxygen species production, and NF-κB-dependent inflammasome priming [19,47,58]. These regulatory connections are supported in broader neuroimmunology. In RP, transcriptomic enrichment of stress response and inflammasome-related programs suggests that p53 may lower inflammatory thresholds under chronic stress. Nevertheless, the precise stage-specific contribution of p53 to inflammasome activation in retinal cell types has not yet been definitively resolved.

### 4.4. A Conceptual p53-Centered Inflammatory Death Circuit

Metabolic stress, ferroptotic susceptibility, microglial activation, and inflammasome priming appear mechanistically interrelated in RP. Rather than asserting a deterministic cascade, we propose a conceptual framework in which p53 may coordinate these processes across photoreceptors and glial populations.

Within this framework, inherited mutations and chronic metabolic strain activate p53 signaling in neurons and glia. In photoreceptors, p53-regulated redox and iron pathways may lower the threshold for ferroptosis. Ferroptosis-associated danger signals engage microglia, whose activation is directly supported in RP models and human tissue.

In microglia, p53-regulated stress programs intersect with NF-κB signaling and inflammasome priming, supporting sustained production of IL-1β, TNF-α, and reactive oxygen species. Evidence supports inflammasome priming signatures; however, the degree of pyroptotic execution in vivo remains uncertain.

Müller glial gliosis and metabolic reprogramming are well documented in RP. By contrast, cell-type-specific p53 roles in Müller glia are supported primarily by indirect transcriptomic enrichment and extrapolation from broader neuroimmunological literature [22,43,44]. Direct causal dissection in RP-specific conditional models is currently limited.

**Figure 3:**
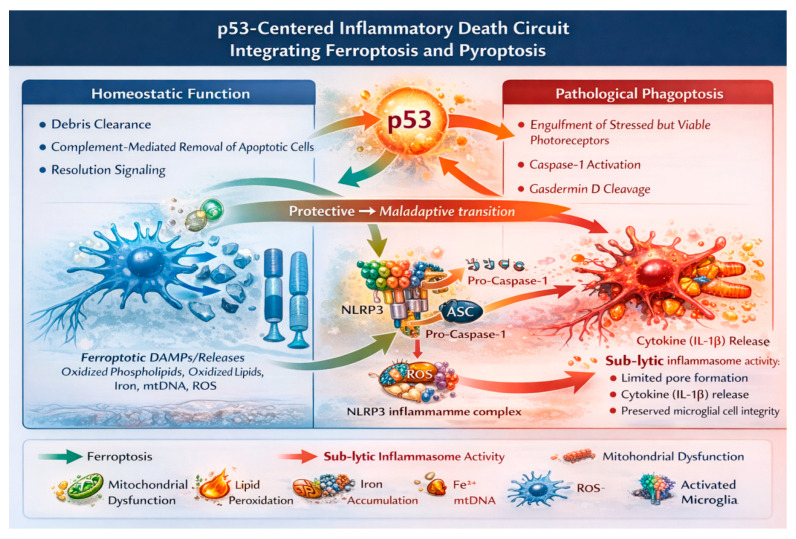
p53-centered inflammatory death circuit integrating ferroptosis and inflammasome-driven neuroinflammation. Schematic illustrating a p53-driven inflammatory circuit that links metabolic stress, regulated cell death, and innate immune activation in retinitis pigmentosa. In photoreceptors, p53-mediated repression of antioxidant pathways lowers the threshold for lipid peroxidation and increases ferroptotic susceptibility. Ferroptosis-associated danger signals—including oxidized Phosphlipids, oxidized lipids, mtDNA, ROS, and iron-related cues—activate microglia and promote inflammatory polarization, metabolic reprogramming, and intracellular iron accumulation. BioRender: https://www.biorender.com (Figures created using the BioRender web-based platform; accessed January 2026.); Adobe Illustrator: Adobe Illustrator 2021 (Version 25.4.1, Adobe Inc., San Jose, CA, USA).

Through iterative interactions—neuronal ferroptotic stress, immune amplification, and glial dysfunction—the retinal environment may shift toward persistent inflammatory pressure. In this formulation, p53 is best viewed not as a unifying executor but as a stress-responsive checkpoint that may modulate the coupling between ferroptosis and inflammasome signaling (Figure 3). Importantly, the evidentiary strength supporting individual components of this model is not uniform. Robust experimental data from RP animal models and human retinal tissue substantiate early microglial activation, complement-dependent phagoptosis, ferroptotic susceptibility in photoreceptors, and inflammasome priming signatures. In contrast, direct causal roles of p53 in Müller glia, the precise extent of gasdermin-mediated pyroptotic execution in vivo, and stage-specific modulation of p53 across retinal cell types remain incompletely resolved and are supported primarily by indirect transcriptomic evidence or extrapolation from broader neuroimmunological contexts. Accordingly, the p53-centered inflammatory death circuit should be interpreted as a hypothesis-generating integrative framework rather than a fully validated mechanistic pathway. From a translational perspective, it suggests potentially mutation-independent immunometabolic strategies—such as modulation of ferroptotic sensitivity, adjustment of inflammasome activation thresholds, and recalibration of microglial metabolic programming—yet these remain conceptual avenues requiring rigorous validation in cell-type-specific and temporally controlled experimental systems before therapeutic conclusions can be drawn.

Despite convergent transcriptomic, mechanistic, and cross-disease evidence suggesting coordinated roles of p53-regulated stress pathways across retinal cell populations, cell-type-specific causal validation of p53-mediated cross-cell integration in retinitis pigmentosa remains limited.

Within microglia, p53 intersects with NF-κB signaling to enhance inflammasome priming. Activation of the NLRP3 inflammasome leads to Caspase-1 activation and gasdermin D cleavage, resulting in limited membrane pore formation. At the molecular level, these gasdermin pores permit IL-1β release. At the phenotypic level, pore formation remains sub-lytic, preserving microglial cell integrity and preventing widespread cell depletion. This sub-lytic inflammasome activity sustains persistent inflammatory signaling without triggering overt pyroptotic rupture.

Müller glia respond to chronic cytokine exposure and oxidative imbalance by stabilizing reactive gliosis, further amplifying chemokine production and redox stress across retinal layers. Together, ferroptotic stress amplification and sub-lytic inflammasome signaling form a self-reinforcing, p53-centered inflammatory death circuit that maintains chronic neuroinflammation and drives progressive retinal degeneration.

## 5. Conclusions

Recent evidence supports a reinterpretation of retinitis pigmentosa as a multicellular immunometabolic disorder rather than a strictly photoreceptor-autonomous genetic disease. Experimental and human data indicate that sustained innate immune activation and non-apoptotic regulated cell death programs actively shape disease progression. In this framework, inflammation is a determinant of degenerative trajectory rather than a secondary consequence.

Within this context, p53 functions not primarily as an apoptotic executor but as a regulator of stress integration. By modulating redox homeostasis, mitochondrial integrity, iron handling, and inflammatory signaling across photoreceptors and glial populations, p53 influences both neuronal susceptibility and inflammatory persistence. This systems-level view helps reconcile variable reports regarding p53 activity in RP and situates it within coordinated immunometabolic regulation.

Therapeutic implications follow directly from this model. Approaches focused exclusively on mutation correction or terminal apoptosis blockade address downstream events, whereas mutation-independent stabilization of ferroptotic sensitivity, inflammasome thresholds, and microglial metabolic programming targets upstream regulatory nodes. Such strategies may be particularly relevant in advanced disease, where inflammatory circuits are already established. Importantly, systemic modulation of p53 or broad immunometabolic pathways carries potential risks, including disruption of tumor suppressive functions, impairment of physiological immune responses, and unintended metabolic consequences, highlighting the need for spatially and temporally targeted strategies.

However, reliance on global p53 ablation limits cell type specificity and mechanistic resolution. Conditional and temporally controlled models are required to dissect p53 function in photoreceptors, microglia, and Müller glia, and to determine whether therapeutic modulation should attenuate, fine-tune, or temporally restrict p53 activity at defined disease stages.

Future work should prioritize longitudinal, cell-type-resolved profiling—using single-cell and spatial transcriptomics—to identify early immunometabolic inflection points that precede irreversible neuronal loss.

Overall, the p53-centered inflammatory death circuit provides a molecular framework linking ferroptosis, inflammasome-associated signaling, and glial metabolic adaptation in RP. Targeting regulatory thresholds within this circuit, rather than isolated downstream pathways, may represent a more effective strategy for durable neuroprotection.

## Figures and Tables

**Table 1:** Immunometabolic pathways in RP: cell type, biomarkers, evidence level, and translational implications.

Pathway	Primary Cell Type	Representative Biomarkers	Evidence Type	Potential Intervention Point
Complement-mediated phagoptosis	Microglia	C3, CR3, C1q	Animal models, human retina	Complement inhibition
Ferroptosis	Photoreceptors	Lipid peroxidation, GPX4, SLC7A11	Animal models	Iron chelation, antioxidant modulation
Inflammasome priming	Microglia	NLRP3, IL-1β, caspase-1	Animal models, transcriptomics	Inflammasome threshold modulation
Müller gliosis	Müller glia	GFAP, metabolic coupling markers	Animal models, human tissue	Gliosis modulation
p53 stress programs	Multiple cell types	p53 targets, redox genes	Indirect, transcriptomics, cross-CNS	Conditional modulation strategies

C3, Complement 3; CR3, Complement Receptor 3; C1q, Complement Component 1 q Subunit; GPX4, Glutathione peroxidase 4; SLC7A11, Solute carrier family 7 member 11; NLRP3, NLR family pyrin domain containing 3; IL-1β, Interleukin-1 beta; GFAP, Glial fibrillary acidic protein.

## Data Availability

No new data were created or analyzed in this study. Data sharing is not applicable to this article.

## Abbreviations

The following abbreviations are used in this manuscript:

RP	Retinitis pigmentosa
ROS	Reactive oxygen species
NF-κB	Nuclear factor kappa B
IL-1β	Interleukin-1 beta
IL-6	Interleukin-6
TNF-α	Tumor necrosis factor alpha
NLRP3	NLR family pyrin domain containing 3
GPX4	Glutathione peroxidase 4
SLC7A11	Solute carrier family 7 member 11
ATP	Adenosine triphosphate
GFAP	Glial fibrillary acidic protein
